# Supplementary material for: Investigating USP42 Mutation as Underlying Cause of Familial Non-Medullary Thyroid Carcinoma
Source: Int J Mol Sci. 2024 Jan 26;25(3):1522. doi: 10.3390/ijms25031522 (PMC10855484; doi:10.3390/ijms25031522)
Supplement: Supplementary file 1 [file ijms-25-01522-s001.zip › Figure S1.pdf]

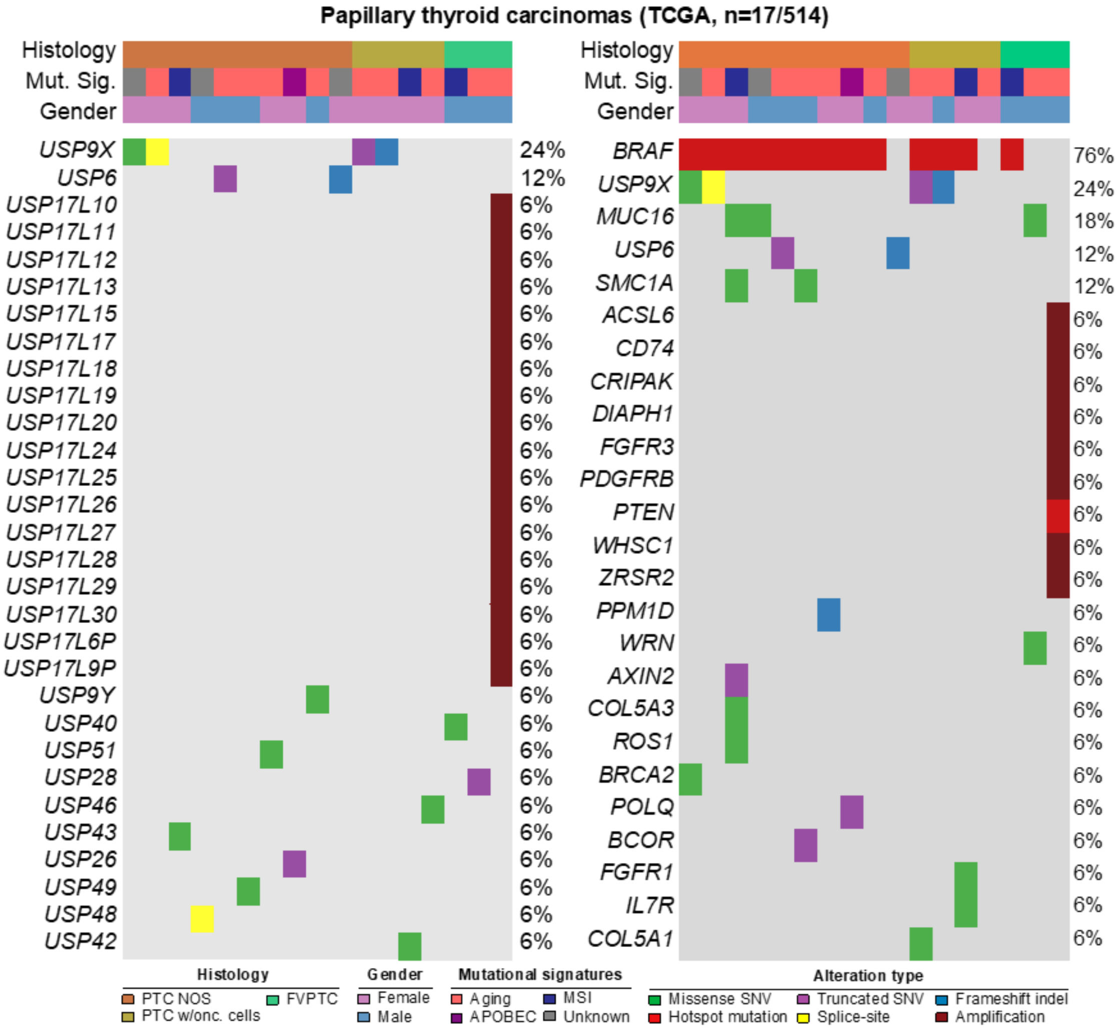

Figure S1 - *In silico* analysis of 514 papillary thyroid carcinomas subjected to whole-exome sequencing from TCGA. Heatmap depicting pathogenic somatic alterations affecting the USP gene family only in papillary thyroid carcinomas (PTC, left), and heatmap depicting all pathogenic somatic alterations affecting the USP-mutant PTCs (right). Cases are shown in columns and genes in rows. Histology, gender, mutational signatures and alteration types are color-coded according to the legend.
