# Supplementary material for: Investigating USP42 Mutation as Underlying Cause of Familial Non-Medullary Thyroid Carcinoma
Source: Int J Mol Sci. 2024 Jan 26;25(3):1522. doi: 10.3390/ijms25031522 (PMC10855484; doi:10.3390/ijms25031522)
Supplement: Supplementary file 1 [file ijms-25-01522-s001.zip › Figure S2.pdf]

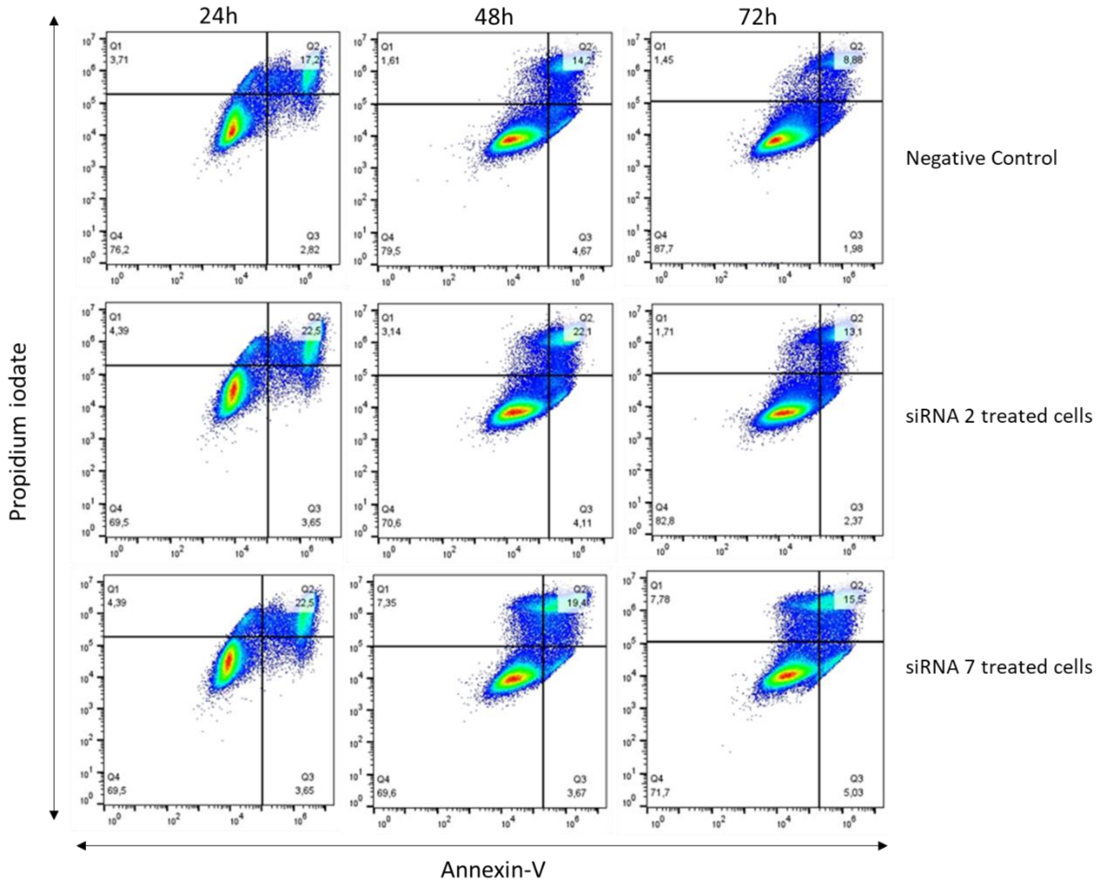

Figure S2 – Apoptosis assays: representative analysis of Annexin-V and Propidium iodate staining. Cell viability and cell mortality analysis at the studied timepoints. Q1 – Necrotic cells (AnnexinV^{-}/PI^{+}), Q2 - Late apoptotic (AnnexinV^{+}/PI^{+}), Q3 - Early apoptotic cells (AnnexinV^{+}/PI^{-}), Q4 – Viable cells (AnnexinV^{-}/PI^{-}). Each experiment was performed with duplicates and three independent experiments were conducted.
