# Supplementary material for: Investigating USP42 Mutation as Underlying Cause of Familial Non-Medullary Thyroid Carcinoma
Source: Int J Mol Sci. 2024 Jan 26;25(3):1522. doi: 10.3390/ijms25031522 (PMC10855484; doi:10.3390/ijms25031522)
Supplement: Supplementary file 1 [file ijms-25-01522-s001.zip › Figure S3.pdf]

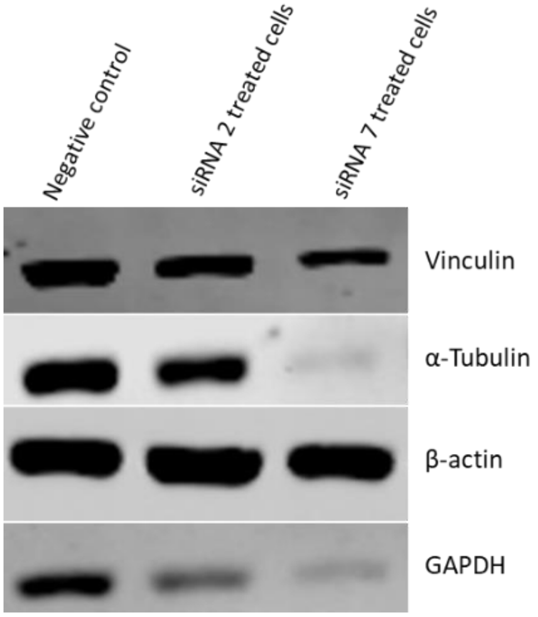


**Figure S3** – Representative blots of housekeeping genes that were silenced by siRNA 2 and 7 treatments. This effect was more evident in siRNA 7 treated cells.
