# Supplementary material for: Investigating USP42 Mutation as Underlying Cause of Familial Non-Medullary Thyroid Carcinoma
Source: Int J Mol Sci. 2024 Jan 26;25(3):1522. doi: 10.3390/ijms25031522 (PMC10855484; doi:10.3390/ijms25031522)
Supplement: Supplementary file 1 [file ijms-25-01522-s001.zip › Table S1.pdf]

Table S1. Somatic pathogenic genetic alterations affecting the UPS gene family in papillary thyroid carcinomas taken from TCGA.

Tumor Sample	Histology	Gene	[1]Chr.	Position/Cytoband	Mutation type	Alternate Allele	Aminoacid change	PolyPhen	SIFT
TCGA-BJ-A3EZ-01	PTC NOS	***USP9Y***	Y	14924964	Missense	A > C	*p.Tyr1529Ser*	possibly_damaging	deleterious
TCGA-CE-A481-01	PTC NOS	***USP6***	17	5076261	Frameshift_Del	TA > -	*p.Met1404Valfs*23*	.	.
TCGA-DJ-A13O-01	FVPTC	***USP40***	2	234468516	Missense	C > A	*p.Asp108Tyr*	probably_damaging	deleterious
TCGA-DJ-A1QE-01	PTC NOS	***USP51***	X	55514742	Missense	G > C	*p.Leu211Val*	possibly_damaging	deleterious
TCGA-DJ-A1QL-01	FVPTC	***USP28***	11	113705033	Nonsense	G > A	*p.Arg187**	.	.
TCGA-DJ-A2Q9-01	PTC with oncocytic cells	***USP46***	4	53492228	Missense	T > C	*p.Gln173Arg*	benign	deleterious
TCGA-E8-A2EA-01	PTC NOS	***USP43***	17	9596525	Missense	C > A	*p.Leu479Ile*	probably_damaging	deleterious
TCGA-EL-A3GP-01	PTC NOS	***USP6***	17	5076150	Nonsense	T > A	*p.Lyr1366**	.	.
TCGA-EL-A3H5-01	PTC NOS	***USP9X***	X	41075213	Missense	A > C	*p.Lys1798Thr*	probably_damaging	deleterious
TCGA-EL-A3MY-01	PTC with oncocytic cells	***USP9X***	X	41043681	Frameshift_Ins	- > A	*p.Pro1105Thrfs*4*	.	.
TCGA-EL-A3ZO-01	PTC NOS	***USP26***	X	132161783	Nonsense_Mutation	G > A	*p.Gln156**	.	.
TCGA-EL-A4K7-01	PTC NOS	***USP49***	6	41767589	Missense	C > A	*p.Arg550Leu*	probably_damaging	deleterious
TCGA-EM-A22P-01	PTC NOS	***USP48***	1	22033083	Splice Site	C > G	*p.X697_splice*	.	.
TCGA-EM-A4FM-01	PTC NOS	***USP9X***	X	41058005	Splice Site	T > C	*p.X1535_splice*	.	.
TCGA-ET-A39P-01	PTC with oncocytic cells	***USP42***	7	6189833	Missense	C > A	*p.Pro669Gln*	benign	deleterious
TCGA-FE-A22Z-01	PTC with oncocytic cells	***USP9X***	X	40988337	Nonsense	G > T	*p.Glu61**	.	.
TCGA-BJ-A0Z0-01	FVPTC	***USP17L10***	4	p16.1	Amplification	.	.	.	.
TCGA-BJ-A0Z0-01	FVPTC	***USP17L11***	4	p16.1	Amplification	.	.	.	.
TCGA-BJ-A0Z0-01	FVPTC	***USP17L12***	4	p16.1	Amplification	.	.	.	.
TCGA-BJ-A0Z0-01	FVPTC	***USP17L13***	4	p16.1	Amplification	.	.	.	.
TCGA-BJ-A0Z0-01	FVPTC	***USP17L15***	4	p16.1	Amplification	.	.	.	.
TCGA-BJ-A0Z0-01	FVPTC	***USP17L17***	4	p16.1	Amplification	.	.	.	.
TCGA-BJ-A0Z0-01	FVPTC	***USP17L18***	4	p16.1	Amplification	.	.	.	.
TCGA-BJ-A0Z0-01	FVPTC	***USP17L19***	4	p16.1	Amplification	.	.	.	.
TCGA-BJ-A0Z0-01	FVPTC	***USP17L20***	4	p16.1	Amplification	.	.	.	.
TCGA-BJ-A0Z0-01	FVPTC	***USP17L24***	4	p16.1	Amplification	.	.	.	.
TCGA-BJ-A0Z0-01	FVPTC	***USP17L25***	4	p16.1	Amplification	.	.	.	.
TCGA-BJ-A0Z0-01	FVPTC	***USP17L26***	4	p16.1	Amplification	.	.	.	.
TCGA-BJ-A0Z0-01	FVPTC	***USP17L27***	4	p16.1	Amplification	.	.	.	.
TCGA-BJ-A0Z0-01	FVPTC	***USP17L28***	4	p16.1	Amplification	.	.	.	.
TCGA-BJ-A0Z0-01	FVPTC	***USP17L29***	4	p16.1	Amplification	.	.	.	.
TCGA-BJ-A0Z0-01	FVPTC	**USP17L30**	4	p16.1	Amplification	.	.	.	.
TCGA-BJ-A0Z0-01	FVPTC	**USP17L6P**	4	p16.1	Amplification	.	.	.	.
TCGA-BJ-A0Z0-01	FVPTC	**USP17L9P**	4	p16.1	Amplification	.	.	.	.

*[1] Chr. – Chomosome; PTC, papillary thyroid carcinoma; NOS, Not otherwise specified; FVPTC, follicular variant of papillary thyroid carcinoma.
